# A Novel Hybrid Membrane for Urinary Conduit Substitutes Based on Small Intestinal Submucosa Coupled with Two Synthetic Polymers

**DOI:** 10.3390/jfb13040222

**Published:** 2022-11-05

**Authors:** Martina Casarin, Martina Todesco, Deborah Sandrin, Filippo Romanato, Andrea Bagno, Alessandro Morlacco, Fabrizio Dal Moro

**Affiliations:** 1 Department of Surgery, Oncology and Gastroenterology, Giustiniani 2, 35128 Padua, Italy; 2 L.i.f.e.L.a.b. Program, Consorzio per la Ricerca Sanitaria (CORIS), Veneto Region, Via N. Giustiniani 2, 35128 Padua, Italy; 3 Department of Industrial Engineering, University of Padua, Via Marzolo 9, 35131 Padua, Italy; 4 Department of Physics and Astronomy ‘G. Galilei’, University of Padova, Via Marzolo 8, 35131 Padua, Italy; 5 Laboratory of Optics and Bioimaging, Institute of Pediatric Research Città della Speranza, 35127 Padua, Italy

**Keywords:** small intestinal submucosa, decellularization, polycarbonate urethane, hybrid membranes, hybrid materials, urinary diversions, regenerative medicine, tissue engineering, biomaterial

## Abstract

Among the urinary tract’s malignancies, bladder cancer is the most frequent one: it is at the tenth position of most common cancers worldwide. Currently, the gold standard therapy consists of radical cystectomy, which results in the need to create a urinary diversion using a bowel segment from the patient. Nevertheless, due to several complications associated with bowel resection and anastomosis, which significantly affect patient quality of life, it is becoming extremely important to find an alternative solution. In our recent work, we proposed the decellularized porcine small intestinal submucosa (SIS) as a candidate material for urinary conduit substitution. In the present study, we create SIS-based hybrid membranes that are obtained by coupling decellularized SIS with two commercially available polycarbonate urethanes (Chronoflex AR and Chronoflex AR-LT) to improve SIS mechanical resistance and impermeability. We evaluated the hybrid membranes by means of immunofluorescence, two-photon microscopy, FTIR analysis, and mechanical and cytocompatibility tests. The realization of hybrid membranes did not deteriorate SIS composition, but the presence of polymers ameliorates the mechanical behavior of the hybrid constructs. Moreover, the cytocompatibility tests demonstrated a significant increase in cell growth compared to decellularized SIS alone. In light of the present results, the hybrid membrane-based urinary conduit can be a suitable candidate to realize a urinary diversion in place of an autologous intestinal segment. Further efforts will be performed in order to create a cylindrical-shaped hybrid membrane and to study its hydraulic behavior.

## 1. Introduction

Bladder cancer (BC) is the tenth most common form of cancer worldwide, with an estimation of 549,000 new cases and 200,000 deaths annually: in males, it is the seventh most commonly diagnosed cancer [1,2]. It is more frequent in men than in women, with incidence and mortality rates among men of 9.5 and 3.3 per 100,000, respectively, which are rates approximately four times higher than those among women globally [3].

Most BCs (70% of cases) are diagnosed as non-muscle invasive (NMIBCs), while the others are muscle-invasive BCs (MIBCs). The guidelines of the European Association of Urology (EAU) classify NMIBCs into low, intermediate, and high risk depending on the treatment options: in the case of low-risk NMIBCs, only transurethral resection of the bladder (TURB) is performed, whereas in the case of intermediate-risk and high-risk NMIBCs, the standard treatment requires TURB with or without Bacillus Calmette–Guèrin (BCG) immune therapy or chemotherapy. A minority of NMIBCs shows unfavorable prognosis, while high-risk NMIBCs have a high rate of disease recurrence and/or progression to muscle-invasive tumors and BCG treatment failure [4].

In the case of MIBCs and in high-risk, unresponsive NMIBCs, radical cystectomy (RC) is performed, creating a urinary diversion using a bowel segment from the patient. However, this surgical approach results in a high risk of complications. The most common ones are associated with the intestinal segment and include strictures, gastrointestinal (GI) tract bleeding, bowel leakage, fistula formations, and the development of metabolic disorders, depending on the dimension of the bowel segment and the type of diversion [5,6,7,8,9].

Therefore, the necessity for an alternative urinary conduit is becoming more and more prominent in urology in order to avoid the use of autologous intestine and reduce the burden of complications. A promising approach can be focused on tissue engineering techniques, which allow largely improving the surgery by reducing its duration, intra-operatory risks and patient complications (e.g., stone formation, urinary tract infections, uretero–enteric strictures, renal function deterioration, and incontinence) [10,11].

Thus far, several types of scaffolds have been investigated, both synthetic and biological, with the aim to identify the best option for urinary conduits. Among synthetic materials, polyglycolic acid (PGA) and poly lactic-co-glycolic acid (PLGA) are in use due to their reliable and reproducible mechanical features, but they often lack sufficient biocompatibility, thus eliciting adverse reactions [12,13,14,15,16]. On the contrary, naturally derived polymers (e.g., silk [17,18], alginate [19], and collagen [20,21,22,23]) showed higher biocompatibility and biodegradability allowing tissue regeneration but with inadequate mechanical properties. A possible disadvantage of biodegradable materials is the degradation rate, which cannot be sufficient to allow tissue reconstruction. For example, this time is estimated at 3–4 months for PGA, 12–16 months for PLA, and 2–4 months for naturally derived polymers such as collagen [24].

Acellular tissue matrices (e.g., small intestinal submucosa (SIS) [25,26,27,28,29,30,31,32], bladder acellular matrix (BAM) [33,34], amniotic membrane (AM) [35], and dermis [36]) preserve the morphology of native tissues. With the aim to preserve the advantages of both natural and synthetic materials and to overcome their limitations, hybrid scaffolds generated by a combination of both types of materials have been recently tested [37].

In the present study, we describe the development of two hybrid membranes; they are obtained by coupling decellularized small intestinal submucosa (SIS) (previously described by our group in [32]) with two commercially available polycarbonate urethanes (Chronoflex AR and Chronoflex AR-LT) with the aim to improve SIS mechanical features. A similar approach was already investigated for the decellularized pericardium intended for cardiovascular applications [38]. The two selected polymers, declared biocompatible and hemocompatible by the producer [39], were compared in combination with decellularized SIS: the hybrid membranes were analyzed to characterize their morphology by immunofluorescence and two-photon microscopy; their mechanical behavior was analyzed through uniaxial mechanical tests; and their cytocompatibility was analyzed through live and dead assays, immunofluorescence, DNA extraction, and metabolic assays.

The results obtained allow hypothesizing the use of the novel hybrid materials for the production of tubular structures to replace urinary conduits.

## 2. Materials and Methods

### 2.1. Porcine SIS Decellularization

Jejunum was collected from an abattoir and treated within 3 hrs. after animal sacrifice. The abattoir’s protocols followed EC guidelines 1099/2009 concerning animal health and protection at the time of sacrifice, was supervised by the Italian government, and was approved by the associated legal authorities of animal welfare (Food and Consumer Product Safety Authority). SIS isolation was obtained accordingly to an already described procedure [32,40,41] by removing the two external muscular layers and serosa and the internal tunica mucosa. Thereafter, SIS was rinsed in phosphate-buffered saline (PBS) and decellularized following the optimized procedure described in [32]. Decellularization is based on the use of protease inhibitors, hypo- and hyper-tonic solutions, Tergitol 15S9 (Sigma-Aldrich, Saint Louis, MO, USA) and Sodium Cholate C1254 (Sigma-Aldrich, Saint Louis, MO, USA), with a subsequent alcohol-based solution. Finally, an endonuclease (Benzonase^®^, E1014, Sigma-Aldrich, Saint Louis, MO, USA) was used to remove DNA residues.

### 2.2. Hybrid Membrane Fabrication

**Figure 1:**
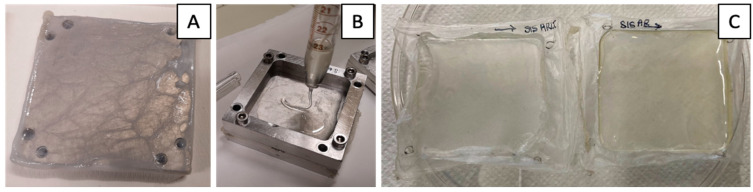
Hybrid membrane production steps: decellularized tissue is gently fixed into the metallic frame (**A**); polymer solution is poured onto the tissue (**B**); and, after 3 days at 38 °C in a vacuum oven, the membranes are removed from the frame (**C**).

Hybrid membranes were produced by solution casting technique and solvent evaporation, following the procedure described by Todesco et al. [38] for pericardium-based hybrid membranes. The main production steps are illustrated in Figure 1. Briefly, decellularized porcine SIS was gently dried with filter paper, then fastened into a customized aluminum frame (50 × 50 mm^2^) with the mucosal side facing down. Therefore, a controlled amount of polycarbonate urethane (PCU) solution (ChronoFlex AR and ChronoFlex AR-LT, AdvanSource Biomaterials, Wilmington, MA, US) was poured on the external side of the SIS. The hybrid membranes and the polymeric membranes used as control in the cytocomptibility tests were dried at 38 °C for 3 days. After desiccation, hybrid and PCU membranes were separated from the frame.

Polymeric membranes are named CF AR (Chronoflex AR) and CF AR-LT (Chronoflex AR-LT) and hybrid membranes are named SIS AR and SIS AR-LT, depending on the PCU. Components of the poycarbonate urethanes can be found in literature: polycarbonate polyol, methylene diisocyanate (MDI), ethylene diamine (EDA), and 1,3-diaminocyclohexane [39]. Chronoflex AR and Chronoflex AR-LT were analyzed in a previous work [38]: Fourier transform infrared (FTIR) spectroscopy confirmed that the two polymers have the same composition since the spectra are largely overlapped. In more detail, the peaks around 1251, 956, and 791 cm^−1^ are due to carbonate groups, and the peak at 1598 cm^−1^ suggests the presence of aromatic molecules. The peak around 1637 cm^−1^ can be related to the diamine content. The signal intensities differ in the range 1200 to 900 cm^−1^: this may be due to the presence of silica microparticles in CF AR-LT, which makes it less sticky. Thermo gravimetric analysis (TGA) confirmed the presence of this inorganic component (~9%).

### 2.3. Immunofluorescence

In order to analyze ECM composition and structural integrity of the SIS after hybrid membrane realization, immunofluorescence was performed following the same procedure that was decribed in our previous paper [32]. For the primary incubation, collagen I (1:100, C2456, Sigma-Aldrich, Saint Louis, MO, USA) and collagen IV (1:200, ab6586, Abcam) were used, while for the secondary incubation Alexa Fluor 555 goat anti-mouse IgG (1:300, A21422, Thermo Fisher Scientific, Waltham, MA, USA) and goat anti-rabbit IgG (1:300, A27039, Thermo Fisher Scientific, Waltham, MA, USA) were applied. Finally, nuclei were showed using DAPI (NucBlue Fixed Cell Stain ReadyProbes reagent, R37606, Thermo Fisher Scientific, Waltham, MA, USA).

For patch immunofluorescence, samples were incubated with phalloidin—Atto 647N (1:200, 65906 Sigma-Aldrich, Saint Louis, MO, USA) and DAPI to stain F-actin and nuclei, respectively.

Leica AF6000 microscope connected to a Leica DC300 digital camera and equipped with LAS AF Software (Leica Micro-Systems, Wetzlar, Germany) was used for image acquisition. Further analysis was performed with ImageJ software.

### 2.4. Two-Photon Microscopy

Decellularized SIS and hybrid membranes (SIS AR and SIS AR-LT) were analyzed with two-photon to evaluate the effect of the hybrid membranes realization procedure on collagen arrangement by assessing the second harmonic generation (SHG) signal [42].

A custom-made multimodal microscope was used [43]. Several z-stacks from decellularized tissue, SIS AR, and SIS AR-LT were acquired with the same settings to make them comparable.

The RAW images were analyzed with ImageJ software [44]. Collagen fiber distribution was evaluated by the application of the fast Fourier transform (FFT) and the coherency (C) parameter: this latter allows estimating the local orientation of the fibers (values around 0 stand for isotropic areas, whereas values around 1 stand for highly oriented structure) using the plug-in OrientationJ [45,46].

Since we wanted to evaluate polymer interaction with decellularized tissue, Dunnett’s multiple comparisons test were made between decellularized SIS, used as control, and both hybrid membranes. The comparison between native and decellularized SIS was previously reported in [32].

During two-photon microscopy analysis, each hybrid membrane sample was excited with a laser at wavelength of 800 nm, and the detector acquired the emitted light in two channels: one at a wavelength of 400 nm, to which the generation of the second harmonic corresponds, as described above, and in which only the collagen is observed. For the second channel, at a wavelength between 435 and 500 nm (blue channel), only the polymer was detected. Finally, SHG and blue channel intensities were calculated by dependence on the depth of the hybrid membranes samples.

### 2.5. FTIR Analysis

In order to check if the polycarbonate urethanes coupled with SIS can alter the secondary structure of tissue proteins and if the polymer penetrates it, hybrid materials were analyzed using the Fourier transform infrared spectroscopy (FTIR). Decellularized SIS and hybrid membranes on the SIS side, and polycarbonate urethanes (CF AR and CF AR-LT) flaps (10 × 10 mm^2^) (n = 3 for each type of material) were equilibrated for 3–4 h. in deuterium oxide (Janssen, Beerse, Belgium). Samples were then processed with a Nicolet iS-50 spectrometer (Thermo Fisher Scientific, Waltham, MA, USA) equipped with the Attenuated Total Reflectance (ATR) accessory. Infrared spectra of the samples and background were recorded using 64 scans in the range of 4000–500 cm^−1^ at room temperature and analyzed with a Matlab^®^ script (MathWorks, Natick, MA, USA) [47].

### 2.6. Mechanical Tests

Dog-bone-shaped specimens of decellularized SIS, SIS AR, and SIS AR-LT were cut according to the ASTM D1708-13 standard (gauge size 5 × 2 mm^2^) [38]. Specimen thickness was measured using a digital caliper (Mitutoyo, model ID-C112XB, Aurora, IL, USA); mean values and standard deviations were quantified.

Uniaxial tensile loading tests were performed by means of a custom-made instrument (IRS, Padova, Italy), which is operated with dedicated software implemented in LabVIEW.

Tests were executed at room temperature, and specimens were kept wet with saline solution. Specimens were pre-loaded up to 0.1 N, then elongated (0.2 mm/s) to breakage. Decellularized SIS, SIS AR, and SIS AR-LT samples were tested along the circumferential and longitudinal directions as described in our previous study [32].

Data were analyzed with an in-house-developed Matlab^®^ script (MathWorks, Natick, MA, USA). Mechanical parameters were quantified from the stress–strain curve plotted for each sample. Ultimate tensile strength (UTS) and failure strain (FS) were calculated as the maximum strength and elongation reached by the specimen, while Young’s modulus (E) was determined as the slope of the stress–strain curves in the linear region.

Statistical tests allow comparing decellularized SIS and hybrid membranes to evaluate the effect of polymers when coupled with the decellularized tissue. The comparison between native and decellularized SIS was previously reported elsewhere [32].

### 2.7. Hybrid Membrane Sterilization

Samples of hybrid membranes (SIS AR and SIS ARLT) were subjected to the sterilization procedure whose efficacy was previously demonstrated [32]. It is based on the use of antibiotics and antimycotics, followed by 0.1% of peracetic acid (PAA).

### 2.8. Sterility Evaluation

Samples (each in duplicate) of sterilized hybrid membranes (SIS AR and SIS AR-LT) were treated according to the European Pharmacopoeia guidelines [48]. Thioglycollate medium (cat no. T9032, Sigma-Aldrich, Saint Louis, MO, USA) and soybean–casein digest medium (cat no. 22092, Sigma-Aldrich, Saint Louis, MO, USA) were used to detect the presence of aerobic/anaerobic bacteria and fungi. Turbidity was visually assessed and images were taken at days 0, 7, and 14.

### 2.9. In Vitro Cytocompatibility Evaluation

The cytocompatibility of SIS, hybrid membrane (SIS AR and SIS AR-LT) and polycarbonate urethane (CF AR and CF AR-LT) samples was evaluated by a direct contact assay, following the part 5 of ISO 10993 [49]. Samples were cut in circular patches (8 mm diameter) and fit into a 48-well plate in aseptic conditions; then, they were incubated with Mesenchymal Stem Cell Growth Medium 2 (cat.no. C-28009, PromoCell, Heidelberg, Germany) containing Supplement Mix (cat.no. C-39809, PromoCell, Heidelberg, Germany) and 1% penicillin–streptomycin at 37 °C. Human mesenchymal stem cells derived from bone marrow (cat.no. C-12974, PromoCell, Heidelberg, Germany) were seeded on the samples at 30,000 cells/cm^2^ and cultured for 1, 7, and 14 days using the abovementioned media. Cell growth was evaluated through immunofluorescence, live/dead staining, metabolic proliferation assay, and dsDNA quantification.

### 2.10. DNA Quantification

DNA content was quantified from decellularized SIS, hybrid membranes (SIS AR and SIS AR-LT) and polymers (CF AR and CF AR-LT) seeded with MSCs after 1, 7, and 14 days using a DNeasy Blood & Tissue Kit (69506, Qiagen, Valencia, CA, USA). Concentration was measured at 260 nm with a NanoDrop One Spectrophotometer (Thermo Scientific, Waltham, MA, USA). Values were normalized for the final volume of the solution to calculate ng of DNA in each sample.

### 2.11. Live/Dead Assays

Cell viability of seeded samples was evaluated after 1, 7, and 14 days with the Live/Dead viability/cytotoxicity kit (MP 03224, Thermo Fisher Scientific, Waltham, MA, USA) to stain live cells in green and dead cells in red. Additionally, the Hoechst 33258 (Sigma-Aldrich, Saint Louis, MO, USA) was used to identify the nuclei. Images were taken with an Olympus IX71 microscope.

### 2.12. WST Assay

Cultured cell proliferation was assessed by WST-1 at days 1, 7, and 14. Absorbance was read at 450 nm with a microplate reader (Spark 10 M Tecan, Tecan, Mannedorf, Switzerland). The values of the non-seeded samples (control) were averaged and taken from the value of each seeded sample.

## 3. Results

### 3.1. Morphology Evaluation of Hybrid Membranes

**Figure 2:**
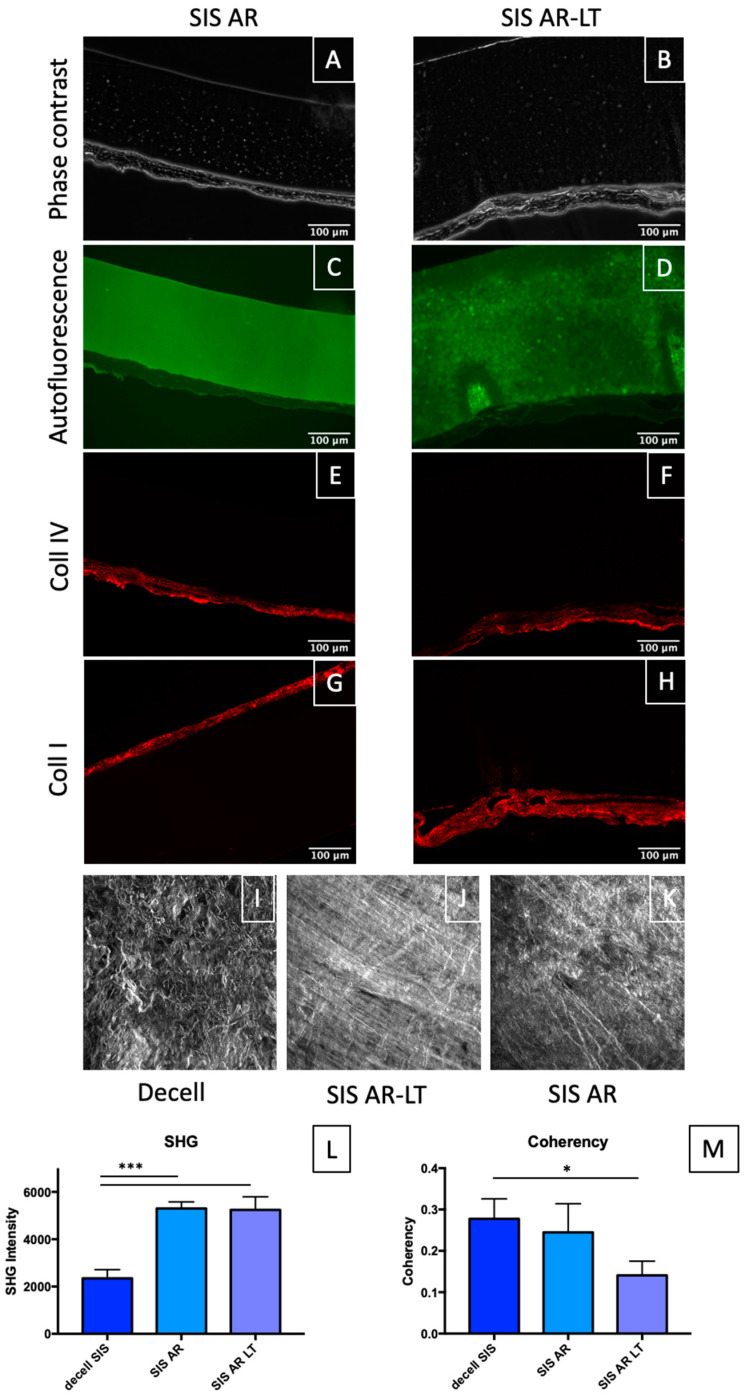
Phase contrast of hybrid membranes: SIS AR (**A**) and SIS AR-LT (**B**). Autofluorescence (green channel) shows the polymers AR (**C**) and AR-LT (**D**), respectively. Collagen IV and collagen I are shown, respectively, in (**E**,**F**) and (**G**,**H**) for both SIS AR and SIS AR-LT. Z-stack of max intensity of phase contrast of decellularized SIS, SIS AR, and SIS AR-LT are shown (**I–K**). SHG intensity values from z-stack measurements (n = 3) (**L**) and coherency analysis from z-stacks (n = 3) (**M**) are presented. Data on histograms show mean ± SD. Data analyzed by Dunnett’s multiple comparisons test (decellularized tissue was used as control group). * *p* < 0.05, *** *p* < 0.001.

With the aim to morphologically characterize SIS AR and SIS AR-LT, phase contrast pictures (Figure 2A,B) and autofluorescence in the green channel (Figure 2C,D) were acquired. Phase contrast shows the distinct layers of polymer (in black) and decellularized SIS (grey levels), while autofluorescence in the green channel better shows the differences between the two polymers: AR (Figure 2C) appears more uniform, while AR-LT (Figure 2D) appears more granular. This evidence can be due to the presence of silica microparticles, which also make AR-LT less tacky than AR. Moreover, in order to evaluate eventual modifications in the decellularized tissue due to polymer coupling, we investigated collagen IV (Figure 2E,F) and collagen I (Figure 2G,H) distribution (they are the main proteins of SIS) in both hybrid membranes. Compared to native and decellularized SIS (data reported in our previous study [32]), collagen I and IV fibers appear more compacted due to the dehydration process necessary to realize hybrid membranes, but no disruption of them can be observed. Similarly, we report the z-stacks of max intensity of phase contrast of decellularized SIS (Figure 2I), SIS AR-LT (Figure 2J), and SIS AR (Figure 2K) to further characterize collagen distribution: collagen fibers appear more stretched in SIS AR and SIS AR-LT compared to decellularized SIS, where they appear more crimped. Consequently, we confirmed the collagen fibers’ denser distribution with SHG intensity calculation, which showed a significant increase following the hybrid membranes realization process (Figure 2L). Instead, coherency parameter calculation showed no significant difference between decellularized SIS and SIS AR but showed a significant difference between decellularized tissue and SIS AR-LT (Figure 2M).

### 3.2. Polymers Penetration in Decellularized SIS

**Figure 3:**
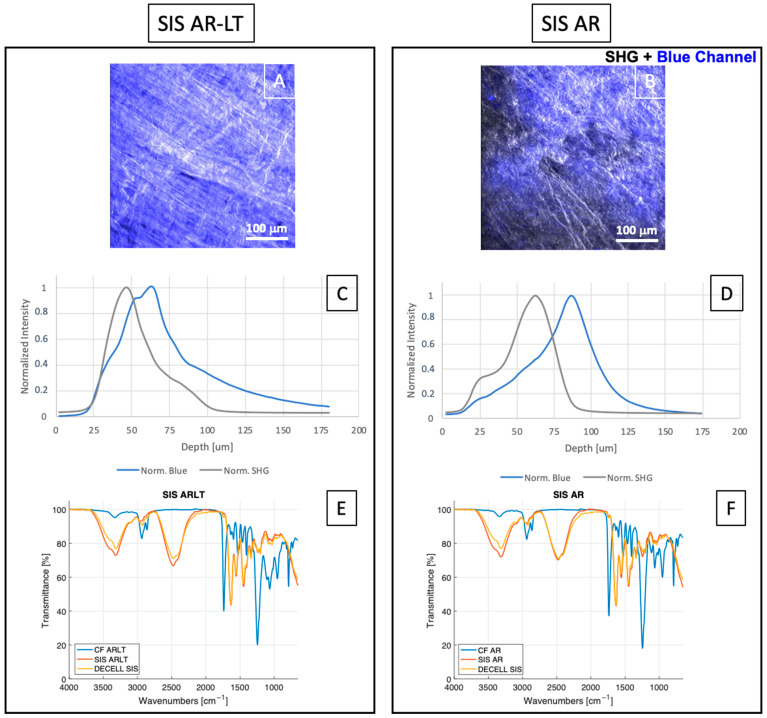
Merge of z-stacks of phase contrast and autofluorescence (blue channel) images (**A**,**B**) of both hybrid membranes. Graphs of normalized intensities of SHG and blue channel for SIS AR-LT (**C**) and SIS AR (**D**). FTIR spectra of decellularized SIS, CF ARLT, and SIS AR-LT (**E**) and decellularized SIS, CF AR and SIS AR (**F**) are reported (n = 3 for each group), showing transmittance (%) over the wavenumbers (cm^−1^).

With the aim to evaluate both polymers’ (CF AR and CF AR-LT) penetration in decellularized SIS, two-photon images of phase contrast and autofluorescence (blue channel) were merged (Figure 3A,B). In Figure 3A, the image of SIS AR-LT is reported, while in Figure 3B the one of SIS AR is reported, showing a good adhesion of both polymeric layers on SIS, with a penetration of CF AR-LT in decellularized tissue higher than CF AR. In Figure 3C,D, the graphs of normalized intensities of blue channel (polymers) and SHG for both hybrid membranes are reported, showing an increased penetration of AR-LT (Figure 3C) in SIS compared to AR (Figure 3D).

Chemical composition was further investigated through FTIR-ATR analysis (Figure 3E,F), where spectra from polymers alone, decellularized SIS, and hybrid membranes were overlapped to highlight similarities. Common peaks are present in both polymers at 1737 cm^−1^ and 1251 cm^−1^: they are specific of the urethane and carbonate groups, respectively. These peaks are not present in the case of decellularized SIS and hybrid membranes. The peak at 1637 cm^−1^ can be due to the presence of a diamine, while the peak at 1598 cm^−1^ is caused by the presence of aromatic molecules in the hard segment of the polymers. Finally, peaks at 1251 cm^−1^, 956 cm^−1^, and 791 cm^−1^ are related to the presence of carbonate groups in the soft segment [38]. Moreover, spectra of the decellularized SIS and hybrid membranes appeared almost overlapped, and typical peaks of protein secondary structure can be detected: common peaks are found at 1631 cm^−1^ and 1558 cm^−1^ due to amide I and amide II binding, respectively. Peaks for collagen are maintained at 1451 cm^−1^, 1339 cm^−1^, 1205 cm^−1^, 1080 cm^−1^, and 1035 cm^−1^, as previously described [32]. These results suggest the maintenance of tissue composition after hybrid membrane fabrication.

### 3.3. Biomechanical Characterization

**Figure 4:**
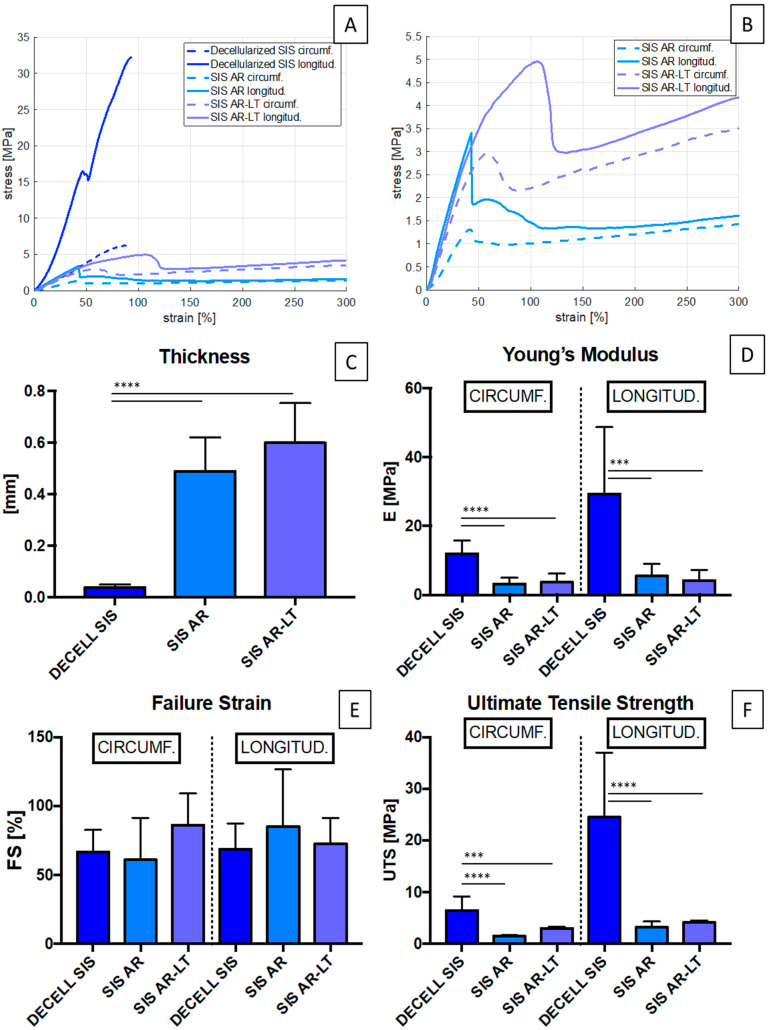
(**A**) Stress/strain graphs (MPa/%) of decellularized SIS and hybrid membranes in circumferential and longitudinal directions. (**B**) Stress/strain curves of hybrid membranes along both directions. Thickness (n = 16), E, UTS, and FS (n = 8) are reported in (**C**–**F**) comparing decellularized SIS and hybrid membranes. A significantly increased thickness is obviously found in hybrid membranes compared to the biological tissue, while a decrease in stiffness and UTS is estimated by Dunnett’s multiple comparison test comparing both membranes to decellularized tissue (control group). No significant FS variation is reported in both membranes compared to the biological tissue, using Dunnett’s multiple comparison test. *** *p* < 0.001 and **** *p* < 0.0001.

Uniaxial tests were performed to compare the mechanical response to load of the decellularized SIS and hybrid membranes (SIS AR and SIS AR-LT): Figure 4A,B show the typical stress–strain curve acquired during the uniaxial tensile test, in which an initial peak due to tissue failure can be distinguished. After this peak, the specimen continues to withstand the elongation due to the presence of the polymer, which prevents the physical breakage of the specimen

With regard to samples thickness, it significantly increased for the hybrid membranes with respect to the decellularized tissue, with a higher increase in the case of SIS AR-LT (Figure 4C). Compared to decellularized SIS, a significant decrease in stiffness (Young’s modulus) (Figure 4D) was observed, both in circumferential and longitudinal directions in SIS AR and SIS AR-LT. The values obtained for decellularized tissue are 11.97 ± 3.8 MPa and 29.33 ± 19.34 MPa along the circumferential and longitudinal directions, respectively, while SIS AR and SIS AR-LT have values of 3.18 ± 1.7 MPa and 3.65 ± 2.5 MPa, respectively, along the circumferential direction and 5.53 ± 3.5 MPa and 4.24 ± 2.9 MPa, respectively, in the longitudinal direction. Moreover, it is worthy to notice that stiffness values became more similar in the two directions, making the mechanical behavior more isotropic.

Similar trends were observed for UTS (Figure 4F) with a significant decrease in mechanical resistance between decellularized tissue and hybrid membranes, which had similar behaviors in both directions: 1.53 ± 0.14 MPa and 3.22 ± 1.1 MPa in the SIS AR circumferential and longitudinal directions, respectively, against 2.99 ± 0.32 MPa e 4.12 ± 0.42 MPa in the SIS AR-LT circumferential and longitudinal directions, respectively.

As for FS (Figure 4E), there is no significant difference between decellularized SIS and hybrid membranes in both directions. In the circumferential direction, SIS AR reaches a lower value (60.88 ± 30.42%) than decellularized tissue (66.64 ± 15.95%), while SIS AR-LT has a greater mean elongation (86.18 ± 22.9%). In the longitudinal direction, SIS AR has a higher mean elongation (85.18 ± 41.4%) than the decellularized tissue (68.63 ± 18.6%), while SIS AR-LT elongates up to 72.61 ± 18.73%.

### 3.4. Sterility Assessment

**Figure 5:**
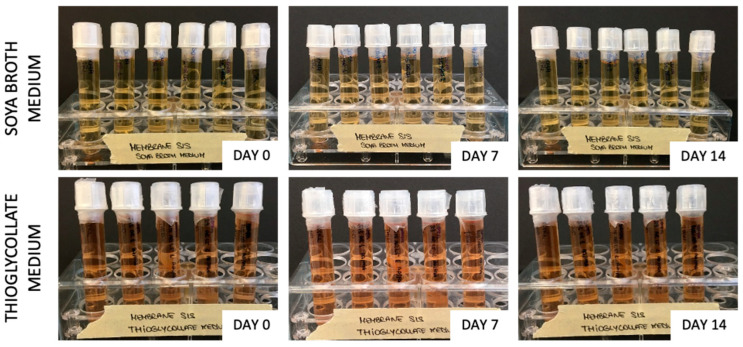
Turbidity tests of SIS AR and SIS AR-LT with thioglycolate medium and soya broth medium.

No turbidity was detected in thioglycolate and soybean–casein digest media broth for SIS AR and SIS AR-LT demonstrating their effective capacity to remove bacteria and fungi (Figure 5).

### 3.5. In Vitro Cytotoxicity

**Figure 6:**
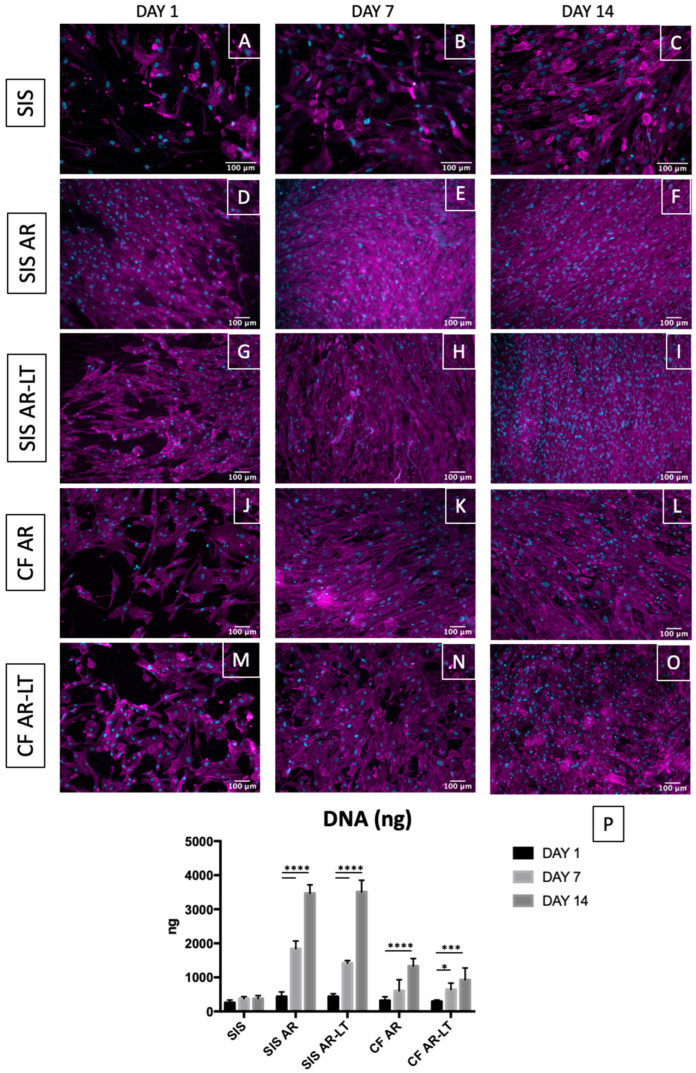
Cell growth on decellularized tissue, hybrid membranes, and polycarbonate urethanes. Phalloidin was used to stain F-actin in magenta and DAPI to stain nuclei in cyan (**A**–**O**). Throughout the experimental period, a gradual cell increasing was observed in all groups, especially in the case of hybrid membranes and polymers alone (here, z-stacks are reported of epifluorescence images). DNA graph (n = 3) (**P**) shows a significant increase between days 1 and 7 and between days 1 and 14 in both SIS AR and SIS AR-LT; between days 1 and 14 in CF AR and days 1 and 7 and days 1 and 14 in CF AR-LT using Dunnett’s multiple comparison test (day 1 of each group was used as control group). No differences were found in the SIS group. * *p* < 0,05, *** *p* < 0.001 and **** *p* < 0.0001. Data show mean ± SD.

Decellularized SIS, SIS AR, and SIS AR-LT from the tissue side and CF AR and CF AR-LT alone were seeded with human bone marrow cells (MSCs) and analyzed after 1, 7, and 14 days from seeding. Nuclei and F-actin were marked with DAPI and phalloidin, respectively (Figure 6A–O). Clear cell growth was demonstrated in each tester: it was more prominent over the hybrid membranes and slightly lower over the polymers. This result was supported by DNA quantification (Figure 6P), which demonstrated an increase of DNA for all samples (SIS, SIS AR, SIS AR-LT, CF AR, and CF AR-LT) from day 1 to day 7 and day 14 but with a more noticeable increase in the case of SIS AR and SIS AR-LT compared to SIS and to both polycarbonate urethanes alone.

**Figure 7:**
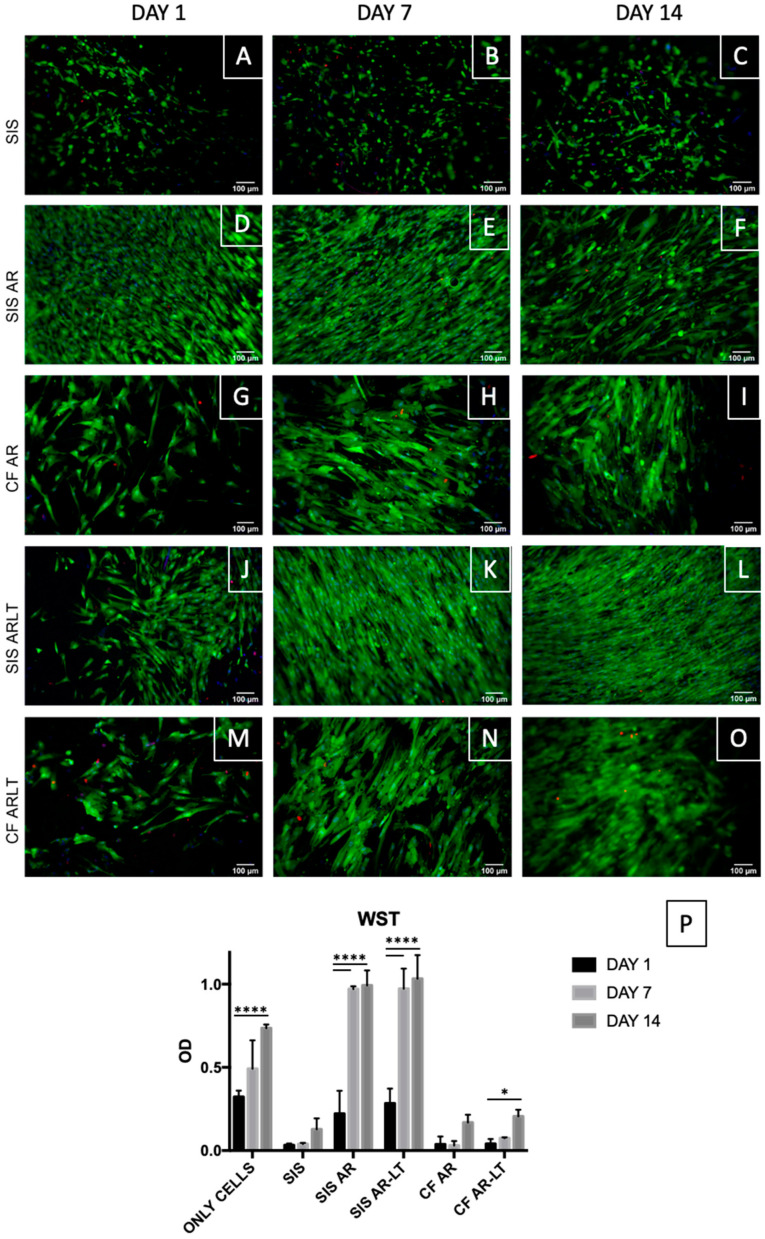
Live/dead staining on seeded SIS, SIS AR, and SIS AR-LT and CF AR and CF AR-LT at days 1, 7, and 14 (**A**–**O**). Limited number of dead cells (in red) were found while an increase in live cells (in green) was found. Nuclei were stained with Hoechst in blue. Optical density values (OD) for SIS, SIS AR, and SIS AR-LT and CF AR and CF AR-LT are reported (n = 3) (**P**) showing a significant difference between days 1 and 14 in control group (only cells) and between days 1 and 7 and days 1 and 14 in both hybrid membranes using Dunnett’s multiple comparison test to compare day 7 and day 14 to day 1 (control). Data show mean ± SD, * *p* < 0,05, **** *p* < 0.0001.

Live/dead staining was performed (Figure 7A–O), confirming the results of fixed cell immunostaining. Viable cells (in green) increased over time from day 1 to day 14 in all groups. On the contrary, very few dead cells were detected over this time frame.

The metabolic activity was quantified in SIS, SIS AR, SIS AR-LT, CF AR, and CF AR-LT seeded with MSCs using the WST-1 assay at days 1, 7, and 14. As control group, cells seeded on plastic were analyzed exhibiting a significant growth between days 1 and 14. Other significant differences were detected between days 1 and 7 and days 1 and 14 in both hybrid membranes and between days 1 and 14 in CF AR-LT, while no significant difference was found in the case of SIS and CF AR. At the same time, both hybrid membranes showed significantly increased values in comparison with cells seeded on plastic, SIS, and polymers alone, especially at days 7 and 14.

## 4. Discussion

The urinary diversion that is usually created after radical cystectomy with a segment of the patient’s bowel causes a number of complications, e.g., strictures, bleeding, leakage, fistula formations, and metabolic disorders [50,51]. In order to prevent these complications, tissue-engineering strategies can be exploited to obtain a urinary conduit characterized by adequate impermeability, patency, compliance, and elasticity [52,53]. Moreover, the engineered conduit has also to allow cell migration, adhesion, proliferation, and differentiation [54].

To date, several approaches have been investigated starting from synthetic materials (e.g., PGA and PGLA) to biological ones (e.g., naturally derived polymers such as silk, alginate, and collagen or acellular tissue matrices such as SIS and BAM, AM, and dermis). However, both kinds of materials showed some limitations, which impaired their application in clinical practice. To overcome these limitations and maintain the advantages of both approaches, the attention has been recently focused on hybrid materials, which combine the typical features of both types of materials. For instance, scaffold obtained from bonding collagen matrix to PGA was proposed for engineering hollow organs such as urinary bladder and it was seeded with urothelial cells (UCs) and bladder smooth muscle cells (SMCs) before implantation in mice [55]. It caused the formation of bladder tissue-like structures consisting of a luminal urothelial layer, a collagen-rich compartment, and a peripheral smooth muscle layer [55]. However, the use of UCs and SMCs is not recommended in oncological applications such as the intended one: therefore, these results are limited to non-oncological cases.

Interestingly, another group created a biodegradable hybrid scaffold consisting of a synthetic polymer (polylactic acid-co-caprolactone, PLACL) and collagen, seeded with neonatal (foreskin) fibroblasts (NNFs) inside and on the top of collagen gels [56]. Similarly, the use of a plastic compressed collagen-polylactic acid-co-ε-caprolactone (PLAC) scaffold for bladder tissue regeneration was investigated [57]. However, also in this study, the authors decided to seed human bladder SMCs and UCs, which were able to proliferate and infiltrate after subcutaneous implantation in the back of nude mice showing an inflammatory reaction lower than PLAC meshes alone. Nevertheless, the use of these types of cells actually impairs the application in oncological patients.

Moreover, another group tested silk biomaterials in combination with extracellular matrix coatings, which were tested with SMCs and UCs, but murine embryonic stem cells (ESCs) and induced pluripotent stem cells (IPSs) were also evaluated for bladder tissue engineering [58]. Additionally, a bi-layered hybrid scaffold was created by electrospinning PLGA microfibers directly onto the abluminal surface of BAM [59]. The scaffold provided good support for growth, attachment, and proliferation of primary bladder smooth muscle cells. After implantation in rats, it was demonstrated the regeneration of bladder tissue structures consisting of urothelium, smooth muscle and collagen-rich layers, which were infiltrated by host cells and micro vessels.

In addition to the choice of the proper cell lines, another critical issue is due to the scaffold porosity: it was demonstrated that in a hybrid scaffold made of BAM and electrospun PLGA microfibers, the increase of the porosity was an effective way to enhance cell proliferation and distribution in vitro and tissue ingrowth in vivo [60]. Similarly, in order to increase porosity, the optimized electrospinning of PLGA was used in combination with plastically compressed collagen scaffolds and minced bladder mucosa to allow in vivo bladder mucosa expansion and support neovascularization followed by tissue ingrowth [61].

Other groups coupled porous extracellular matrices such as AM or BAM with other biomaterials in order to create novel hybrid materials. For instance, amniotic membrane was used in combination with PLCL and implanted in rats [62],. Similarly, BAM was used in combination with degradable polyester urethane (PEU) and PLGA by directly electrospinning, which was successfully implanted in rats, [63]. More recently, a conductive bio composite was created combining graphene with AM for the replacement of the neuronal network of tissue-engineered urinary bladder [64]. Additionally, in this case, the scaffold was seeded with SMCs and UCs, but the electrical stimulation applied in vitro led to an increased SMCs growth and linear arrangement.

Following these results, we decided to use decellularized SIS for urinary conduit reconstruction in our previous study [32]: it was reported to be a porous scaffold that favors cell adhesion and proliferation [65]. We were able to effectively decellularize porcine SIS conduit by using an optimized protocol that allowed maintaining natural ECM components, thus demonstrating SIS cytocompatibility with human fibroblasts. Unfortunately, decellularized SIS showed limited impermeability and mechanical resistance, which can affect its suitability as urinary conduit substitute due to the cytotoxic effects of urine on cells and the inability to keep the conduit patent. Other groups tried to solve this problem by testing different types of materials. For instance, a very innovative scaffold obtained from a decellularized squid mantle was recently presented to create an impermeable tissue-engineered urinary conduit [66]. Unfortunately, the authors used a Triton X-100-based decellularization protocol whose toxicity has been recently ascertained [67]. In addition, they seeded UCs and SMCs on decellularized squid mantle, which consequently limits its application to only non-malignant cases.

In order to overcome the impermeability issue, another group tested a tissue-engineered tubular polypropylene mesh scaffold, which was first pre-implanted before the ureter reconstruction model in pig [68]. However, this procedure requires longer time before ureter reconstruction.

In the present study, we decided to keep decellularized SIS in order to exploit its biocompatibility, but improving its mechanical features and impermeability by creating two types of hybrid membranes (SIS AR and SIS AR-LT) coupling SIS (whose role would be to accommodate a large number of cells) with two polycarbonate urethanes (CF AR and CF AR-LT) which serve as a barrier, as already performed with decellularized porcine pericardium [38]. CF AR and CF AR-LT are two commercially available solutions of polycarbonate urethanes in N,N-dimethylacetamide (DMAc) (22% (*w*/*v*). They are declared biocompatible and hemocompatible and differ for the presence of silica microparticles in CF AR-LT to make the polymer less tacky. Moreover, these polymers were chosen since they are suitable for molding, casting and dip-coating fabrication techniques and can resist stress cracking and exhibit high flexural resistance.

We demonstrated the maintenance of collagen fibers in decellularized SIS after the realization of hybrid membranes through immunofluorescence, and two-photon analysis. In both SIS AR and SIS AR-LT, collagen fibers appeared less crimped and more stretched compared to SIS: this may be due to the realization process of hybrid membranes, which requires SIS dehydration with filter paper to prevent polymer coagulation. Moreover, two-photon and FTIR analyses demonstrated a higher penetration of Chronoflex AR-LT in the SIS in comparison with Chronoflex AR.

Biomechanical results demonstrated a significant decrease of hybrid membrane stiffness with a reduction of Young’s modulus (E) and ultimate tensile strength (UTS) in both circumferential and longitudinal directions. Correspondingly, the maximum elongation achieved by the materials (FS) in both directions was not significantly different from that of the decellularized tissue. Generally, the mechanical behavior of the hybrid membranes appeared more isotropic in comparison with decellularized SIS, with less prominent differences between the two directions. This can be a key feature for the ideal urinary conduit, which must resist physiologic hydraulic pressures without permanent deformations.

Prior to biocompatibility evaluation, it was crucial to confirm the sterilization effectiveness after the realization of hybrid membranes. For this reason, we applied the same chemical sterilization procedure that was demonstrated to be effective in the case of the decellularized SIS [32]: it was based on the use of a mix of antibiotics/antimycotics and peracetic acid. Following the protocol provided by the European Pharmacopoeia [48] for turbidity tests, we were able to demonstrate the efficacy of this sterilization procedure since turbidity did not appear even after 14 days.

Cytocompatibility was then tested by direct contact assay to study attachment, migration and distribution of the seeded cells within the scaffold. We decided to use human MSCs isolated from bone marrow in order to study an adequate cell type to be applied also in cases of malignancy [69,70] and able to differentiate into other specific cell types such as UCs and SMCs [71,72,73], which is in agreement with Kloskowski et al. [74], who concluded that the most appropriate cells source are bone marrow-derived MSCs. Moreover, we avoided the use of fibroblasts as in our previous study [32] since Drewa [75], who used SIS in combination with 3T3 fibroblasts for urinary diversion in rats, concluded that this type of cell cannot serve as a “feeder layer” for ureteral augmentation. The experiment was performed with MSCs over both hybrid membranes and over the control groups (decellularized SIS and only polymers). In the light of this comparison, we were able to appreciate a significant improvement in the number of MSCs grown on hybrid membranes compared to decellularized SIS alone: cells strongly adhered and grew on SIS AR and SIS AR-LT over time, showing a parallel increasing in the metabolic activity. This result was observed also for the polymers alone (CF AR and CF AR-LT), but it was less prominent than for hybrid membranes: polymers may facilitate cell growth due to the increased impermeability compared to the SIS alone. Therefore, hybrid membranes do improve cytocompatibility and significantly promote MSCs proliferation over time with respect to the decellularized SIS and polymers alone.

The results obtained suggest the adequacy of both hybrid membranes to realize a novel urinary conduit.

## 5. Conclusions

SIS-based hybrid membranes have shown to be a promising scaffold for tissue engineering applications and for urinary conduit substitution. The realization of hybrid membranes did not interfere with the decellularized SIS collagen content (which is the main tissue component), but polymers changed the mechanical properties of the biological tissue, decreasing its stiffness and leveling the mechanical behavior along the circumferential and longitudinal directions. Moreover, the presence of the polymer did not weaken SIS cytocompatibility, but rather it significantly improved them, allowing greater cell growth and organization over time compared to the decellularized SIS alone.

In order to satisfy the intended clinical need, the future developments of this research will be to realize a conduit made of hybrid membrane which is repopulated with specific cell types and to pre-clinically evaluate it through in vivo studies on animal models.

## Data Availability

Not applicable.
